# Keratinocytes maintain compartmentalization between dermal papilla and fibroblasts in 3D heterotypic tri‐cultures

**DOI:** 10.1111/cpr.12668

**Published:** 2019-08-05

**Authors:** Justin J. Y. Tan, John E. Common, Chunyong Wu, Paul C. L. Ho, Lifeng Kang

**Affiliations:** ^1^ Department of Pharmacy National University of Singapore Singapore Singapore; ^2^ Skin Research Institute of Singapore Singapore Singapore; ^3^ Department of Pharmaceutical Analysis China Pharmaceutical University Nanjing China; ^4^ School of Pharmacy University of Sydney Sydney NSW Australia

**Keywords:** 3D culture, hair follicle, hydrogel, keratinocytes, tissue engineering, tri‐culture

## Abstract

**Objectives:**

Reproducing human hair follicles in vitro is often limited by various reasons such as the lack of a systematic approach to culture distinct hair follicle cell types to reproduce their spatial relationship. Here, we reproduce hair follicle‐like constructs resembling the spatial orientation of different cells in vivo, to study the role of keratinocytes in maintaining cellular compartmentalization among hair follicle‐related cells.

**Materials and methods:**

Dermal papilla (DP) cells, HaCaT keratinocytes and human dermal fibroblast (HDF) cells were seeded sequentially into three‐dimensional (3D) microwells fabricated from polyethylene glycol diacrylate hydrogels. Quantitative polymerase chain reaction was used to compare inductive gene expression of 3D and two‐dimensional (2D) DP. DP and HaCaT cells were transfected with green fluorescent protein and red fluorescent protein lentivirus, respectively, to enable cell visualization using confocal microscopy.

**Results:**

The 3D DP cultures showed significantly enhanced expression of essential DP genes as compared 2D cultures. Core‐shell configurations containing keratinocytes forming the outer shell and DP forming the core were observed. Migratory polarization was mediated by cell‐cell interaction between the keratinocytes and HDF cells, while preserving the aggregated state of the DP cells.

**Conclusions:**

Keratinocytes may play a role in maintaining compartmentalization between the DP and the surrounding HDF residing in the dermis, and therefore maintains the aggregative state of the DP cells, necessary for hair follicle development and function.

## Graphical abstract

Cultures comprising of dermal papilla cells, keratinocytes, and human dermal fibroblasts were prepared from sequential seeding into hydrogel microwells, in order to reproduce the spatial orientation of hair follicular cells in vivo. This work demonstrated the importance of keratinocytes in maintaining cellular compartmentalization and dermal papilla aggregation, which is necessary for hair follicle development and function.

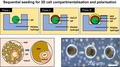

## 1 INTRODUCTION

The hair follicle is often considered a mini organ residing in the dermal layer of the skin. It is a composite of various specialized and differentiated cells, with each residing in a distinct compartment and having unique functional roles that regulate hair growth, mediated by a series of complex cell‐cell interactions.^1^ Research concerning the hair follicles has established several findings essential for understanding their biology necessary for recapitulating microenvironments suitable for de novo hair follicle neogenesis. Firstly, it was established that the dermal papilla (DP) cells express greater in vivo like character in their gene and protein expression when cultured in three‐dimensional (3D) microenvironments as compared to flat two‐dimensional (2D) surfaces. This is because evidences have suggested that when the DP is removed from their in vivo microenvironment to culture in a 2D environment, DP cells gradually lose their inductivity for the hair cycle development.^2^ However, they are able to partially restore this inductivity when transferred from 2D to 3D cultures.^3^, ^4^, ^5^ Secondly, 3D co‐cultures of DP and keratinocytes have been investigated for their roles in hair follicle formation and cycling, which are mostly regulated by a series of sequential reciprocal interactions between these epithelial and mesenchymal cells.^6^, ^7^ Co‐grafted dissociated epithelial and dermal cells from mice reposition themselves spontaneously to reproduce their anatomical relationship in vivo, implying the significance of this positional relationship between epithelial and mesenchymal cells in the outcomes of follicular morphogenesis.^8^ Therefore, strategies have been introduced to achieve cellular compartmentalization and findings have supported the notion that optimizing cell positional relationships can help promote appropriate epithelial‐mesenchymal interaction (EMI) for hair follicle bioengineering. Pan et al fabricated micro‐structured poly (ethylene glycol) diacrylate (PEGDA) hydrogels resembling the physiological architecture of hair follicle, in which the dermal cells were encapsulated within the gel compartment, separated from the epidermal cell populations.^9^ Similarly, Lim et al developed a method to assemble DP and normal human epidermal keratinocytes (NHEK) in close proximity within 3D fibrous hydrogel scaffolds using two oppositely charged polyelectrolyte solutions.^10^ This method encourages cells cultured within one domain to establish interactions among themselves, and with the second cell type in the neighbouring domain, thereby improving EMI. Yen et al developed a protocol based on the differential adherence of keratinocytes and DP cells to an ethylene vinyl alcohol (EVAL) surface, producing hybrid cell spheroids having preferential compartmental core‐shell structure, with an aggregated DP cell core surrounded by a keratinocyte shell.^11^ This method enables self‐assembly of the DP cells and keratinocytes to form a core‐shell structure, which partially resembles the cell compartmentalization in vivo, instead of randomly distributing the cells within co‐cultured spheroids. Limitations of the study included that the cells used were from rats and the sizes of self‐assembled cell spheroids were very variable. Present studies have only investigated the roles of the DP cells and the keratinocytes as important for the development of the hair follicles, notwithstanding the importance of their behaviour towards other cell types, such as the human dermal fibroblast (HDF). The HDF constitutes an essential component of the skin as they contribute to the extracellular matrix of the dermis and communicate with each other and other cell types, playing a crucial role in regulating skin and hair follicle physiology.^12^

Polyethylene glycol (PEG) is a synthetic polymer advantageous for 3D cultures owning to its hydrophilicity, relative inertness, relatively low protein adsorption and amenability to user‐defined crosslinking chemistry and presentation of ligands to cells.^13^ In addition, it has the capability for photopolymerization, adjustable mechanical properties, and easy control of scaffold architecture and chemical compositions.^14^ PEG hydrogels have also been commonly used in repairing and regenerating a variety of tissues due to its biocompatibility and non‐immunogenicity.^14^ A typical method of crosslinking PEG chains is by the photopolymerization of diacrylate‐terminated PEGDA monomers under ultraviolet (UV) irradiation.^15^ PEGDA hydrogels can be micro‐moulded to form microwells in a consistent and high‐throughput manner via soft lithography, the fabrication of replicas using elastomeric polydimethylsiloxane (PDMS) stamps.^9^, ^16^, ^17^ Micro‐moulding allows the shape, size and geometry of hydrogel scaffolds to be modified to mimic in vivo conditions.^16^ In our study, PEGDA hydrogels patterned with 200 μm microwells were fabricated as the diameter of the hair bulb was shown to be around 150‐200 μm from histological images.^18^ Herein this study, we introduce a systematic approach for producing 3D core‐shell heterotypic spheroids of controlled sizes by the sequential seeding of dissociated human DP cells, keratinocytes and HDF cells into microarray hydrogels fabricated from PEGDA using soft photolithography. Preparing the heterotypic spheroids in this way restores the spatial orientation of these cells in order to investigate the effects on their cell compartmentalization. In this study, we have observed that keratinocytes may have a role in maintaining the aggregative state of the DP cells within the dermis, which could be necessary for the hair follicle development and function.

## 2 MATERIALS AND METHODS

### 2.1 Materials

Dulbecco's modified Eagle's medium (DMEM), foetal bovine serum (FBS), random primers and SYBR safe DNA gel stain were supplied by Invitrogen and Life Technologies. Trypsin and penicillin/streptomycin solution were obtained from PAN‐Biotech GmbH. RNeasy Mini Kit and QuantiFast SYBR Green PCR kit were purchased from Qiagen. Random primers and avian myeloblastosis virus reverse transcriptase were purchased from Promega. All other reagents were of analytical grade obtained from conventional commercial sources and used as supplied.

### 2.2 Master fabrication

Photomasks were designed using AutoCAD 2010 and printed on chromium coated soda lime glasses at Infinite Graphics PTE LTD. Silicon wafers were spin‐coated with negative photoresist SU‐8 2075 (MicroChem Corp.) at 167.7 *g*, yielding the desired film thickness about 200 µm. Wafers were soft‐baked at 65°C for 7 minutes followed by a second soft‐baking at 95°C for 60 minutes. For crosslinking of the photoresist, the coated wafers were exposed to UV light of 350‐400 nm for 90 seconds through the photomask by using a single‐side mask aligner (SVC, Model H94‐25). Subsequently, the wafers were post‐exposure baked at 65°C for 6 minutes and then at 95°C for 15 minutes. The photoresist‐patterned silicon masters were developed using SU‐8 developer, rinsed with isopropyl alcohol for 10 seconds, and air‐dried with pressurized nitrogen.

### 2.3 PDMS stamp fabrication

Prepolymer siloxane elastomer base solution was mixed with curing agent Sylgard 184 (Dow Corning Corporation) at a 10:1 ratio by mass. The PDMS prepolymer mixture was poured onto a silicon master with a SU‐8 photoresist coating patterned with an array of 200 µm microwells and degassed for 20‐25 min in a vacuum chamber to remove any air bubbles before curing at 70°C for 2 hours. The PDMS layer was peeled off from the silicon master and cut to a suitable size. The resulting PDMS stamp had patterns corresponding to the silicon master in the form of micropillars with diameters of 200 µm each and was imaged using a stereomicroscope (Nikon SMZ25).

### 2.4 PEGDA microwell array fabrication

UV‐photocrosslinkable PEGDA (Jenkem Technology) of molecular weight 3500 Da were mixed with photoinitiator Irgacure 2959, HHEMP (Sigma‐Aldrich) and diluted with 1xPBS to form a prepolymer solution comprising of the photoinitiator. The patterned PDMS stamp was placed on an evenly distributed film of prepolymer solution on a TMSPMA (Sigma‐Aldrich)‐treated cover slip, with two coverslips placed on both sides as spacers. Photopolymerization was achieved by irradiating the set‐up with UV light of 320‐500 nm and at an intensity of 4.96 W/cm^2^ for 30 seconds using the OmniCure^®^Series 2000 curing station (Lumen Dynamics) as previously optimized. After photopolymerization, the PDMS stamp was peeled from the fabricated hydrogel microwell arrays, which were submerged in 70% ethanol for 2 hours to remove excess prepolymer solution. Hydrogel microwell arrays were subsequently washed thrice with PBS and stored in sterile PBS under aseptic conditions prior to cell seeding.

### 2.5 Cell culture

The immortalized DP cell line was donated by Professor Mike Philpott and Dr Adiam Bahta from Queen Mary University London for this work. The cell lines were previously isolated and immortalized from DP cells obtained from scalp biopsy.^19^ The HDF and HaCaT cells are gifts from the Institute of Medical Biology, Agency for Science, Technology and Research (A*STAR). DP, HaCaT keratinocyte cells and HDF were manipulated under aseptic conditions and maintained in a humidified incubator at 37°C with 5% CO_2_ atmosphere. Media components were filtered through 0.22 μm pore Corning filter units (Corning Incorporated). Culture media consisted of DMEM (Invitrogen Corporation) supplemented with 10% FBS (Invitrogen Corporation, USA), 1% 10 000 U/mL penicillin and 10 mg/mL streptomycin (PAN‐Biotech GmbH).

### 2.6 Preparation of 2D cultures

DP cells, HaCaT keratinocytes and HDF cells were cultured in T75 flask until ~80%‐90% confluency. Then, the cells were trypsinized and counted. Each cell type was then centrifuged and reconstituted with fresh culture media to a concentration of 500 000 cells/mL. A total of 150 µL from each cell type were used in the preparation of co‐cultures or tri‐cultures and were seeded into a 35‐mm glass‐bottomed Petri dish and observed on day 1 and day 4.

### 2.7 Preparation of 3D cultures

3D tri‐cultured spheroids were prepared by seeding DP cells, followed by HaCaT keratinocytes and finally HDF cell suspension in this sequence, respectively, into the microwells, with a 24 hours time duration between each seeding, by a previously reported wiping method.^20^ The concentration of cells used for the seeding was as follows: 12 million cells/ml for DP and 24 million cells/ml for HaCaT and HDF cells. Briefly, 15 µL of cell suspension was pipetted along the edge of a microscope glass coverslip which was then slowly wiped across a microwell array. 3D co‐cultured spheroids were prepared using seeding densities of 12 million cells/mL for DP and 24 million cells/ml for HaCaT or HDF cells. Sequential seeding was performed at 24 hours interval between each seeding. The same wiping technique was used as described above.

### 2.8 Visualization of cell distributions in 2D and 3D cultures

Lentiviral supernatant is produced by co‐transfecting HEK293T cells with pLenti‐GFP [or pLenti‐RFP)] and ViraPower™ Lentiviral Expression System (Invitrogen) according to the manufacturer's instructions. DP and HaCaT cells were seeded at 50 000 cells/well in a 35 mm culture dish, respectively, and incubated overnight. DP and HaCaT cells were infected with GFP and RFP lentivirus, respectively, for 48 hours. GFP‐expressing DP cells and RFP‐expressing HaCaT cells were sorted and isolated from non‐transfected cells using flow cytometry with the instrument Beckman Coulter Moflo Astrios. Positively transfected cells were cultured and used in the preparation of 2D and 3D cultures using the method as described above. These cells, including the HDF cells (neither expressing GFP nor RFP) were then counterstained with the blue fluorescent DAPI nucleic acid dye (Invitrogen).

### 2.9 mRNA extraction and quantitative real‐time PCR

mRNA was extracted from cells using the RNeasy Mini Kit (Qiagen) in accordance with the manufacturer's instructions. Reverse transcription of total mRNA was performed using random primers and avian myeloblastosis virus reverse transcriptase. Quantitative real‐time PCR reaction was performed using Rotor‐Gene Q real‐time PCR cycler (Qiagen). Primers (Integrated DNA technologies, Singapore) used for PCR reactions were listed in Table 1. The primer sequences were designed using Primer3 (http://frodo.wi.mit.edu/) and Primer‐BLAST (http://www.ncbi.nlm.nih.gov/tools/primer-blast/). β‐actin primer was included as an internal loading control. Each reaction mixture was prepared using 10 μL QuantiFast SYBR Green PCR master mix, 4 μL of cDNA template with 1 μmol/L of each primer in a total reaction volume of 20 μL. The PCR was run for 40 cycles, and the thermal cycling conditions were as follows: initial heat activation at 95°C for 10 minutes; denaturation for 10 seconds at 95°C; combined primer annealing and extension for 60 seconds at 60°C. The fluorescence signal was measured at the end of each extension step. After the amplification, a melting peak analysis with a temperature gradient from 72°C to 95°C was performed. Fluorescence emission readings were analysed using Rotor‐Gene Q software (Qiagen). The data were presented as the fold increase in the target gene expression, normalized to the housekeeping gene β‐actin.

**Table 1:** DNA sequence of primer pairs used for quantitative real‐time PCR

Gene symbol	Forward primer (5′‐3′)	Reverse primer (5′‐3′)
β‐actin	TGGCATCCACGAAACTACCT	CAATGCCAGGGTACATGGTG
AXIN2	AGGGAGAAATGCGTGGATAC	CTGCTTGGAGACAATGCTGT
BMP2	ATCACGCCTTTTACTGCCAC	TAGCACTGAGTTCTGTCGGG
BMP4	AAGCTAGGTGAGTGTGGCAT	CGAGATAGCTTGGACGGGAA
BMP6	AGCATAACATGGGGCTTCAG	GAAGGGCTGCTTGTCGTAAG
FGF7	CACACAACGGAGGGGAAATG	GCCATAGGAAGAAAGTGGGC
FGF10	TCCTCCTCCTTCTCCTCTCC	TAGCTTTCTCCAGCGGACAT
HEY1	ATACGCCTGCATTTACCAGC	TCAATTGACCACTCGCACAC
Noggin	GACGGGGGAACTTTTGTAGA	CTTCGAGGTCCAAGGAAAAC
SFRP2	TTCCCCAAGCACACTCCTAG	CAAGATTCGGGTGGGCTTTT
SOSTDC1	TGGAGGCAGGCATTTCAGTA	CACACACCAGCTCCTTCAGA
LEF1	TTATCCCTTGTCTCCGGGTG	ATAGCTGGATGAGGGATGCC
WNT5A	CGTTAGCAGCATCAGTCCAC	TGTGCCTTCGTGCCTATTTG

### 2.10 Statistical analysis

Results were expressed as means ± standard deviation of at least three independent experiments. Statistical analysis was performed by Student's *t* test. The difference was statistically significant at *P*‐value < .05.

## 3 RESULTS

### 3.1 PEGDA hydrogel microarray fabricated using soft lithography

**Figure 1:**
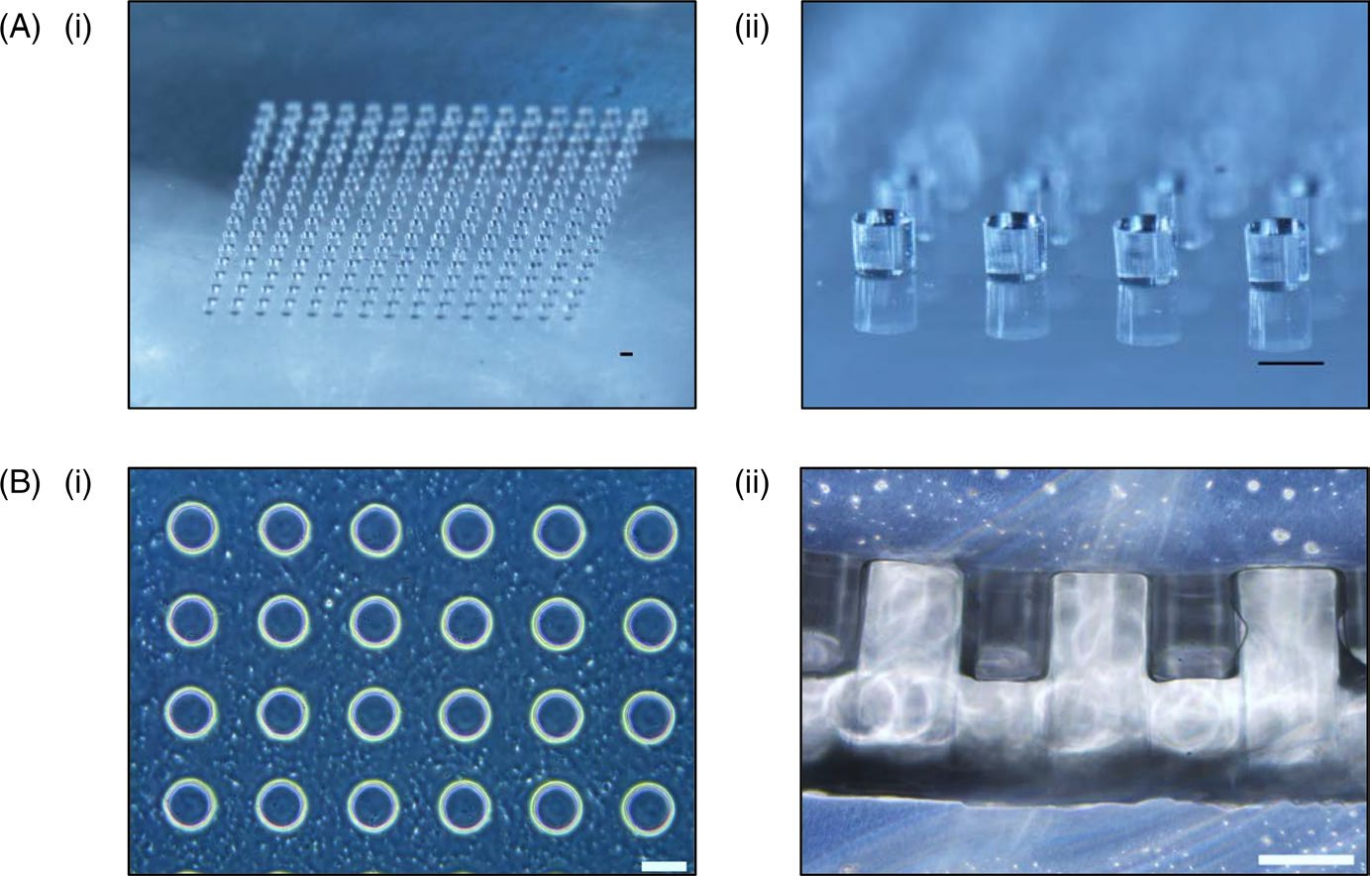
Fabrication of PEGDA microwell arrays. A, Images of PDMS mould fabricated by soft lithography on a micropatterned silicon wafer. (i) 15 by 15 array displaying PDMS micropillars. (ii) Close‐up image of the PDMS micropillars. For each PDMS micropillar, diameter = height = 200 µm. B, Images of the PEGDA microwell array. (i) Close‐up image showing the top view of the PEGDA microwell array. (ii) Cross‐section of the PEGDA microwell array, where diameter of each microwell = height of each microwell = 200 µm. (Scale bar = 200 µm)

The PDMS stamp, as shown in Figure 1A(i) and (ii), was fabricated using a silicon master with SU‐8 photoresist coating patterned with microwells according to the steps outlined in Tan et al^20^ The PEGDA hydrogel microwell arrays were then fabricated on top of the 3‐(Trimethoxysilyl)propyl methacrylate (TMSPMA) treated coverslips, using PDMS stamp as a mould via UV initiated crosslinking of diacrylate groups, from prepolymer solution containing 5% w/v PEGDA MW3500 and 0.09% w/v of the 2‐hydroxy‐4′‐(2‐hydroxy‐ethoxy)‐2‐methylpropiophenone (HHEMP) photoinitiator. Using the PDMS stamp as a mould, microwells with diameter of 200 µm each were formed as shown in Figure 1B(i) and (ii). Each PEGDA hydrogel microarray contained 15 by 15 microwells, hence allowing the seeding and subsequent formation of 225 aggregates.

### 3.2 3D tri‐cultures produced with sequential seeding

**Figure 2:**
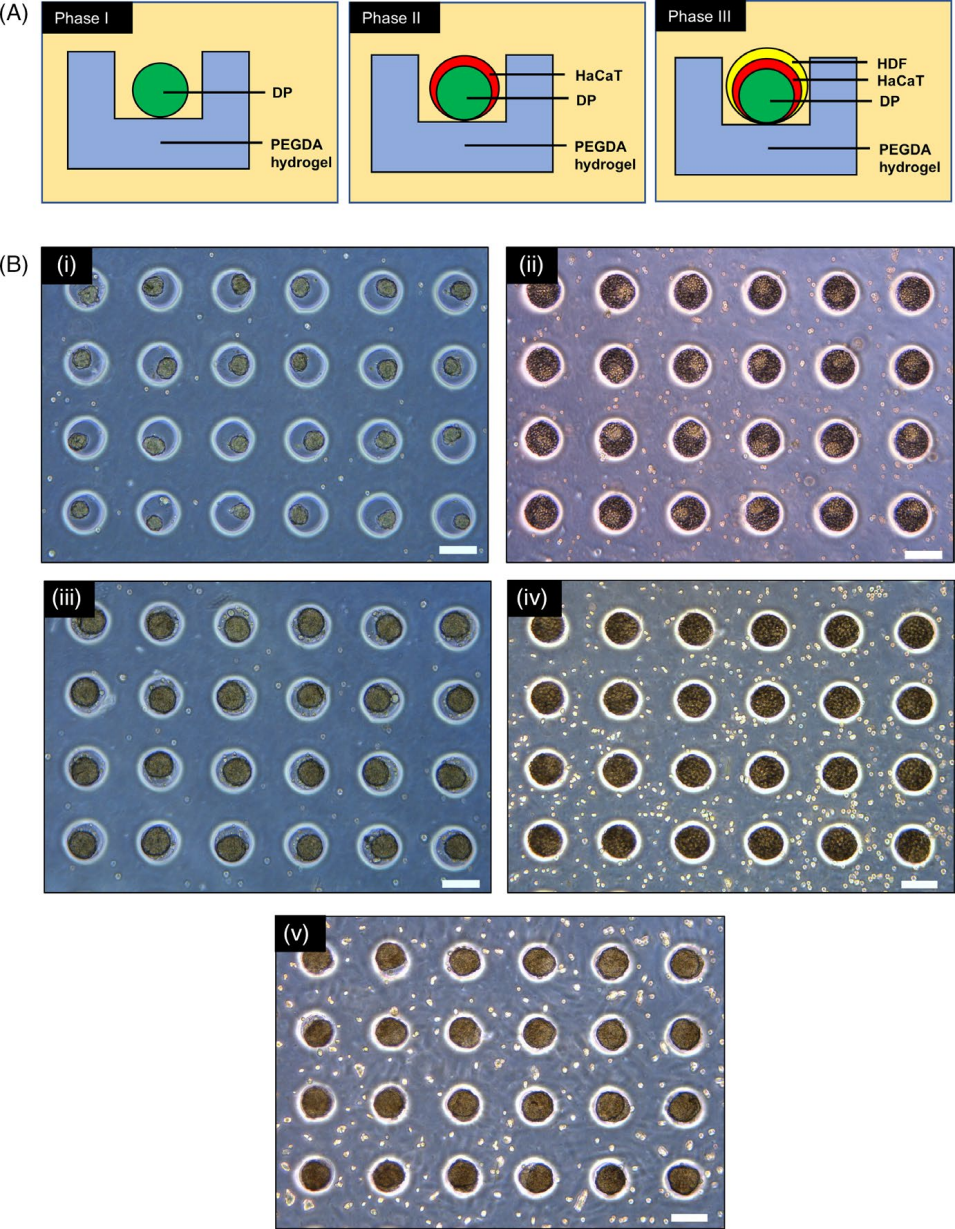
Preparation of 3D tri‐cultures by sequential seeding. A, Schematic representation of the sequential seeding approach, a process of sequentially seeding cells of a different type on top of another to achieve compartmentalization. The phases indicate the stages of cell seeding to yield 3D co‐cultures and tri‐cultures, respectively. B, Illustration of sequential seeding using light microscopic images. (i) DP aggregates formed 24 h after seeding into empty PEGDA microwells at 12 million/mL. (ii) DP aggregates were seeded with HaCaT keratinocytes at 24 million/mL. Outlines of the DP aggregates can be observed. (iii) 24 h after seeding of HaCAT keratinocytes onto DP aggregates to form DP‐HaCaT co‐cultured aggregates. (iv) HDF cells were seeded at a concentration of 24 million/ml above DP‐HaCaT co‐cultured aggregates. (v) 24 h after seeding of HDF at a concentration above DP‐HaCaT co‐cultured aggregates to yield DP‐HaCaT‐HDF tri‐cultures. All scale bars represent 200 µm

Human DP cells, human adult low calcium high temperature (HaCaT) keratinocytes and HDF were cultured and compartmentalized in the core‐shell configuration, with the DP cell aggregate forming the core and the HaCaT keratinocytes/HDF forming the shell. Sequential seeding enabled the DP cells to form spherical aggregates 24 hours post‐seeding in the microwell array, after which HaCaT keratinocytes and subsequently HDF cells were seeded sequentially above the DP aggregates to yield co‐cultures or tri‐cultures. Figure 2A illustrates the proposed arrangements of different cell types in relation to each other upon their sequential seeding into the microwells to yield the core‐shell configuration. We have further demonstrated, using green fluorescent protein‐expressing DP cells and red fluorescent protein‐expressing HaCaT keratinocytes to show that the core‐shell structure is indeed achievable using our PEGDA microwell constructs. As the HDF we used neither expresses GFP nor RFP, regions stained with blue DAPI nuclei dye which does not produce any red or green fluorescence were taken to be HDF and their cell behaviours were studied.

The preparation of co‐cultured and tri‐cultured aggregates was illustrated using light microscopic images as shown in Figure 2B**.** PEGDA offers a low attachment surface to the cells, hence promoting cellular aggregation. This encouraged the formation of 3D DP spheroids 24 hours post‐seeding into the microwells. (Figure 2B(i)) The formation of DP spheroids enabled compartmentalization, allowing HaCaT keratinocytes to be seeded above them to produce DP‐HaCaT co‐cultured aggregates at 24 hours post‐seeding of the HaCaT keratinocytes. (Figure 2B(iii)). Similarly, HDF cells can be seeded above the DP‐HaCaT co‐cultured spheroids to produce tri‐cultured spheroids as illustrated in fure 2B(v).

### 3.3 3D cultures enhance expression of DP inductive genes in DP aggregates

**Figure 3:**
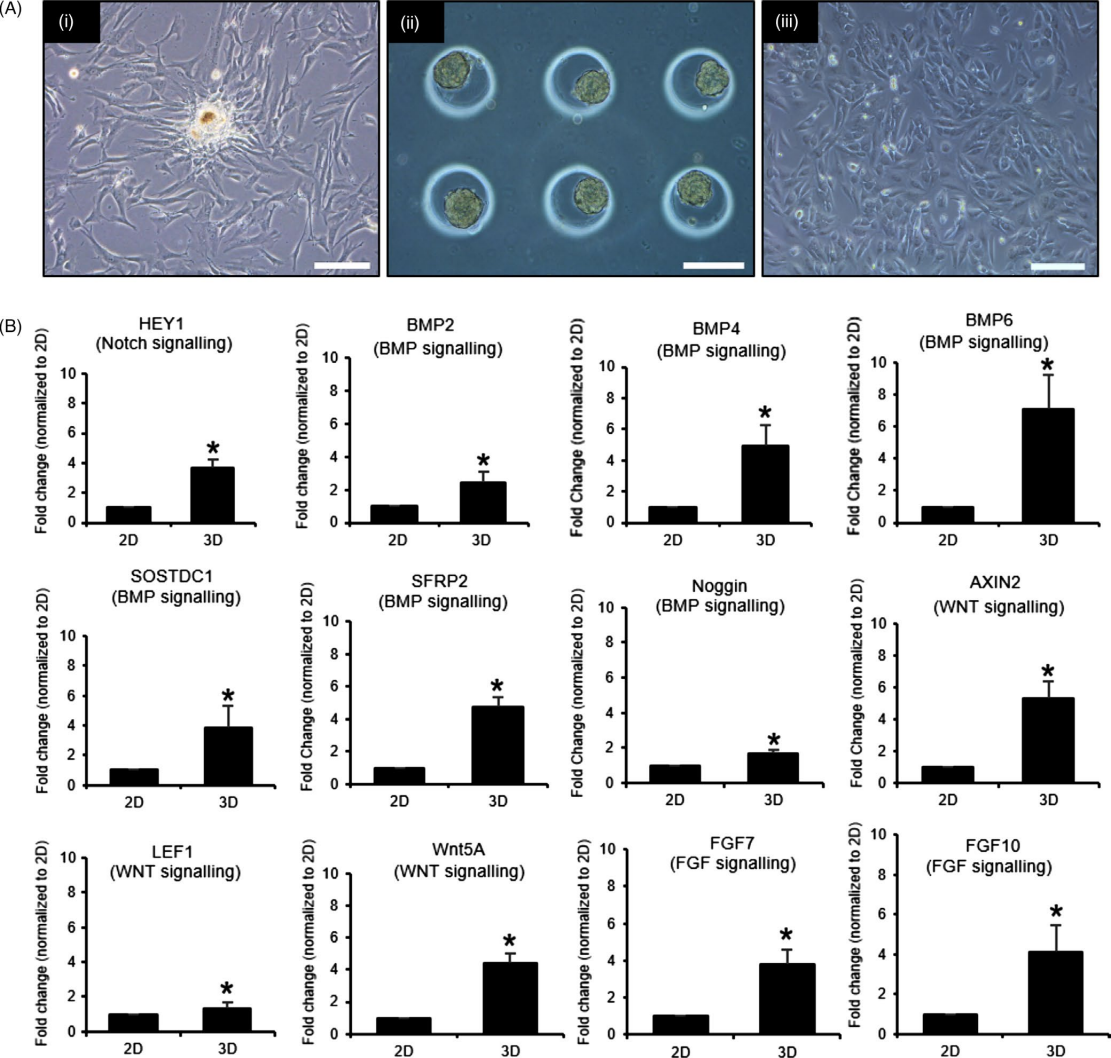
Aggregation improves expression of DP inductivity genes, making them more in vivo like. A, Photograph illustrations showing (i) aggregation among early isolated primary human DP is a native character of the DP (ii) Immortalized DP restored to its aggregative state in 3D cultures (iii) Immortalized DP in late passages of 2D cultures does not exhibit aggregative behaviour. B, 3D immortalized DP mono‐cultures upregulates expression of genes necessary for in vivo DP hair inductivity as compared to immortalized DP cells cultured on flat 2D Petri dish after 48 h. All scale bars represent 200 µm

Early isolated cultures of primary DP exhibited signs of intercellular aggregation as shown in Figure 3A(i). To recapitulate the intercellular aggregation between the DP cells, immortalized DP cells were seeded into the microwells, upon which 24 hours post‐seeding undergoes aggregation to produce 3D DP aggregates as shown in Figure 3A(ii). These microwells facilitate intercellular aggregation for late passage immortalized DP, as shown in Figure 3A(iii), which do not exhibit aggregation in 2D culture. Figure 3B demonstrated that 3D mono‐cultures of immortalized DP showed significantly enhanced expression of essential DP genes responsible for its inductivity as compared to immortalized DP cells cultured on flat 2D surfaces. These genes included pathway ligands and inhibitors such as HEY1 (from the Notch signalling), BMP2, BMP4, BMP6, SOSTDC1, SFRP2 and Noggin (from the BMP signalling), AXIN2, LEF1, WNT5A (from the WNT signalling), FGF7 and FGF10 (from the FGF signalling).

### 3.4 Spontaneous rearrangement of cells yields core‐shell aggregates in 2D co‐cultures

**Figure 4:**
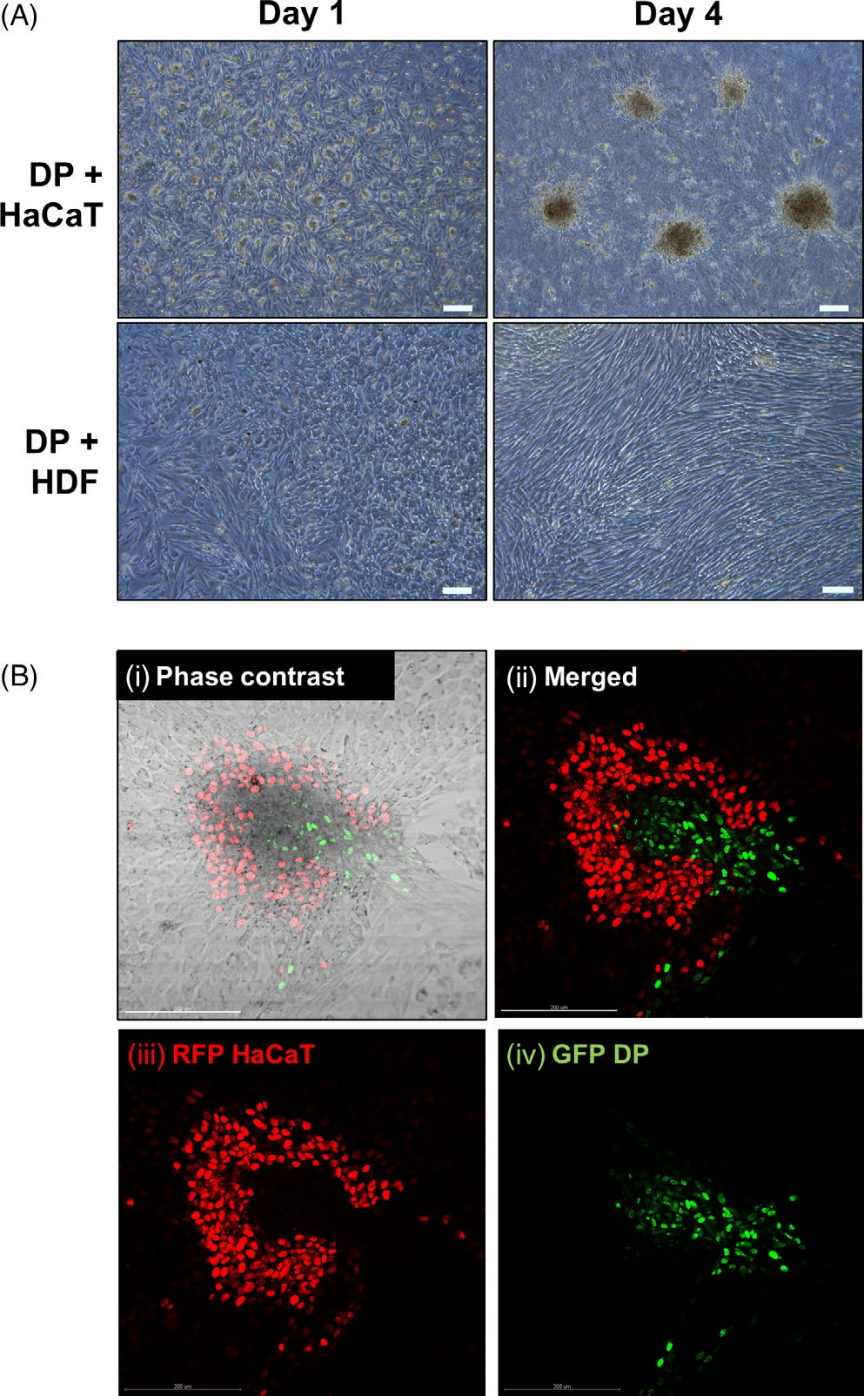
2D distributions of DP cells when co‐cultured with HaCaT keratinocytes or HDF. A, Aggregation of DP cells observed when DP is seeded together with HaCaT keratinocytes on a Petri dish, but not when DP is seeded together with HDF on day 4 of co‐culture. B, Profile of the aggregates observed in DP‐HaCaT 2D co‐cultures. (i) Phase contrast image of a single aggregate (ii) Merged fluorescent image showing the GFP transfected DP aggregate surrounded by RFP transfected HaCaT cells (iii) RFP transfected HaCaT cells forming a shell configuration (iv) GFP transfected cells forming a core aggregate configuration. All scale bars represent 200 µm

Distributions of DP cells co‐cultured with HaCaT or HDF cells were investigated when these cells were co‐seeded together onto 2D Petri dishes for a duration of 1 and 4 days. Green fluorescent protein (GFP)‐expressing DP cells and red fluorescent protein (RFP)‐expressing HaCaT cells were later used to determine their relative positions on the 4th day of incubation. It was observed that aggregates were forming on the 4th day of co‐incubating the DP cells and the HaCaT keratinocytes, but not when the DP cells were co‐incubated with HDF. (Figure 4A) The profile of the aggregates observed in the DP and HaCaT keratinocyte co‐cultures were determined using GFP‐expressing DP cells and RFP‐expressing HaCaT keratinocytes. Figure 4B(i)‐(iv) illustrates that DP and HaCaT cells in 2D cultures were spontaneously rearranging themselves to produce aggregations of core‐shell configurations of DP cells surrounded by HaCaT keratinocytes.

Similarly, keratinocytes are known to exhibit similar behaviour in 2D tri‐cultures (comprising of DP, HaCaT and HDF), which is to facilitate the aggregation of DP cells, an essential feature for restoring DP inductivity and function. Similar core‐shell configurations were observed (Figure S1), with the keratinocytes forming the shell and the DP aggregates forming the core.

### 3.5 Migratory polarization of cells observed within 3D tri‐cultures

**Figure 5:**
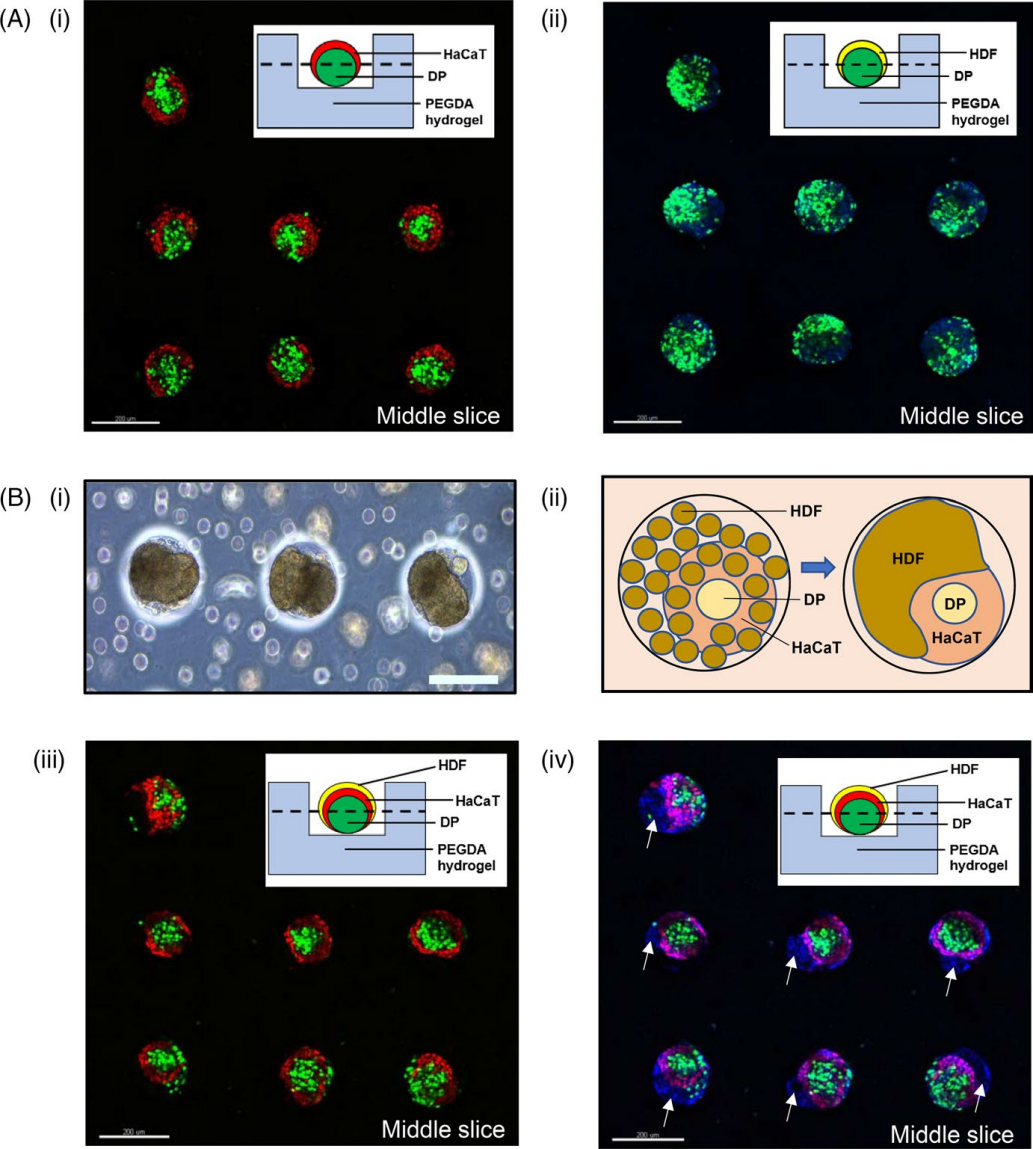
Polarization of cells observed in 3D tri‐cultures but not in co‐cultures at 24 h. A, Confocal images of (i) middle slice of the 3D microwells containing GFP‐expressing DP surrounded by RFP‐expressing HaCaT. (ii) middle slice of the 3D microwells containing GFP‐expressing DP dispersing throughout HDF (stained with blue nuclei DAPI dye). B, Images of 3D DP‐HaCaT‐HDF tri‐cultures: (i) Light microscopic images (10x magnification) of 3D tri‐cultures showing cell polarization. (ii) Schematic diagram postulating the HDF polarizing from DP‐HaCaT co‐cultured aggregate. (iii) Middle slice of the confocal image showing just the RFP‐expressing HaCaT surrounding GFP‐expressing DP. (iv) Middle slice of the confocal image showing 3D tri‐cultured aggregates with white arrows indicating regions where HDF have polarized and migrated away from DP‐HaCaT aggregates. These regions are neither stained red or green, indicating the absence of either DP or HaCaT cells. All scale bars represent 200 µm

Similar core‐shell configurations were reproduced in 3D co‐cultures of DP‐HaCaT spheroids prepared from the sequential seeding of these cells inside the microwell arrays as observed using confocal laser scanning microscopy. Figure 5A(i) and (ii) illustrates the middle slices of the DP‐HaCaT spheroids and DP‐HDF spheroids, respectively. From the 3D reconstructions of the image stacks in Figure 5A(i), it was observed that (a) GFP‐expressing DP cells were present as aggregates in the mid‐sections of the image stacks; (b) RFP‐expressing HaCaT cells remained primarily surrounding the DP aggregates at the sides of the image stacks. On the other hand, Figure 5A(ii) shows that the GFP‐expressing DP cells did not remain in its aggregated state when co‐cultured with HDF cells (stained with blue nuclei dye) but were dispersed throughout the HDF cells when cultured in 3D microwells. The core‐shell configurations of the DP‐HaCaT spheroids were preserved within the 3D tri‐cultured spheroids, while it was observed that signs of migratory polarization of the HDF cells were present, as observed in Figure 5B(i). It was observed that the migratory polarization was mediated by cell‐cell interaction between the HaCaT keratinocytes and the HDF cells, while preserving the aggregated state of the DP cells, as illustrated by the diagram in Figure 5B(ii)‐(iv) clearly illustrates the role of the HaCaT keratinocytes in maintaining compartmentalization of the DP cells and the HDF cells, as we observed that only the HaCaT keratinocytes (ie RFP‐expressing HaCaT keratinocytes) were directly interacting with HDF cells (which did not express any RFP or GFP and thus was only stained blue by the 4',6‐diamidino‐2‐phenylindole (DAPI) dye), by contrasting the image of Figure 5B(iii), which only showed the RFP‐expressing HaCaT keratinocytes and the GFP‐expressing DP cells, with Figure 5B(iv), which showed both the RFP‐ and GFP‐expressing cells with HDF cells. The white arrows thus indicate the presence of HDF cells polarizing away from the DP‐HaCaT co‐cultured spheroids.

## 4 DISCUSSION

The human hair follicle contains both the mesenchymal and ectodermal components.^21^ The ectodermal component in the hair follicle is characterized by the invagination of the epidermis into the dermis and contributes the hair matrix which gives rise to the hair shaft. Surrounded by these ectodermal structures is the DP, which is a small assembly of specialized fibroblasts regulating the hair cycle and is also the mesenchymal component of the hair follicle. The interactions between DP and epithelial cells in the hair follicle have been known to be essential for the development and growth of the human hair.^22^ HaCaT keratinocytes are spontaneously immortalized human keratinocyte cell line which has been used in the studies of epidermal homeostasis and pathophysiology.^23^ The HaCaT cell line is known for its high differentiation potential in cell culture due to its expression of several epidermal differentiation markers. As such, the HaCaT cell line, as the ectodermal keratinocytes and one of the functional components of the hair follicle structure has been used in the study of drug effects on the hair follicle, including inflammation and proliferation.^21^, ^24^

In this work, we have established that keratinocytes may also have a role in maintaining the compartmentalization of the DP cells from the surrounding HDF cells within the dermis. The presence of tight cell‐cell contacts between keratinocytes may be the reason for inhibiting DP migration across the epithelial sheet, thereby maintaining the aggregation of the DP within the native hair follicle. Evidence of epidermal tight junctions was highlighted in previous publications.^25^, ^26^ Based on current understanding, there have been no cell culture models to demonstrate the function of epidermal tight junctions in maintaining cellular compartmentalization. HDF, on the other hand, does not have tight cell‐cell contacts, which permits the migration of DP cells across gaps between HDF cells and therefore, unable to maintain the aggregation of DP necessary for maintaining its inductivity and function. Evidence of the absence of tight junctions is highlighted in a previous publication.^27^

The role of keratinocytes in ensuring that the DP cells remains in its aggregated state is important as aggregation is known to be an essential feature of DP inductivity. Adult rodent DP has been shown to induce de novo hair follicle development and hair growth when they are excised and transplanted into non‐hair bearing recipient skin.^28^ These imply that the DP has the capacity to reprogram non‐hair‐bearing epidermis to a follicular fate, repeating the events of embryonic hair morphogenesis. The inductivity potential of the DP cells was preserved in early rodent DP cell cultures as they were able to induce new hair follicle formation and subsequent hair fibre development in recipient epithelium.^29^, ^30^, ^31^

However, despite the extraordinary inductive capabilities of murine DP in reconstitution assays, human DP cells are less susceptible to induce hair follicle neogenesis.^32^ These differences were highlighted between species‐specific DP, in which cultured rodent DP form papilla‐like aggregates within the dermis after transplantation, but not cultured human DP.^29^ Therefore, it was believed that the aggregation of DP is necessary for hair follicle development because their early dispersal leads to a disruption of hair follicle formation.^33^ In our study, we have observed clearly that aggregation is necessary for the upregulation of the essential signalling pathways of the DP related to hair follicle inductivity, including the WNT, BMP and FGF signalling pathways, as these were significantly upregulated in the intact DP, as revealed from microarray analysis of freshly micro‐dissected DP.^34^ The genes selected for testing were based on prior publications investigating for specific DP expression and thus, relevant to DP inductivity (ie these genes are responsible for specific DP function to induce new hair growth within the follicles).^28^, ^31^, ^34^ Therefore, the upregulation of these genes is crucial to demonstrate the restoration of DP function when they are cultured in the 3D system.

The effect of HDF on DP inductivity has not been investigated previously primarily because for hair follicle in vivo, the dermal papilla is surrounded by the keratinocytes and is compartmentalized from the surrounding dermal fibroblasts, minimizing their interaction with the DP. In terms of physiological relevance, researchers have shown that keratinocytes play a greater role in maintaining the inductivity of DP as compared to the dermal fibroblasts. Future work may persist in trying to investigate the effects of HDF on DP inductivity. We have also clearly observed that the DP cells do not show signs of aggregation when co‐incubated with the HDF cells, a predominant cell type residing within the dermal layer of the skin, but were mixed homogenously, unlike when the DP cells were co‐incubated with the HaCaT keratinocytes. It can be hypothesized that the formation of aggregates is a process of spontaneous compartmentalization among cells in vivo, allowing for the formation of specialized skin features, such as the hair follicles.

To study these cell behaviours in 3D microenvironments, we fabricated PEGDA microwell arrays using soft lithography. 3D cell cultures offer a more accurate representation of cell polarization, since in 2D, the cells can only be partially polarized.^35^ We have also shown that DP cells cultured in 3D exhibited better gene expression in the signalling pathways relating to hair inductivity, as compared to those grown in 2D.

In addition, with our tri‐culture models, we have discovered that cells cannot be sensibly compartmentalized to mimic the in vivo microenvironment in 2D cultures and the lack of fluorescence markers in HDF means they cannot be objectively identified as compared to GFP‐expressing DP or RFP‐expressing HaCaT cells, while in 3D cultures, sequential seeding enabled the compartment of the cells in the system and therefore, facilitated their identification and behaviour.

By seeding the cells sequentially into low attachment PEGDA microwells, we have established a co‐culture and a tri‐culture system to restore the in vivo spatial orientation of these cells—DP, keratinocytes and HDF. We have observed that DP aggregates exhibited the core‐shell configuration when co‐cultured with keratinocytes in 2D and 3D cultures. The 2D system shows that keratinocytes may have a role to play in establishing DP aggregation, a key feature responsible for restoring DP inductivity and function. This feature is observed in native DP when they are freshly isolated from the hair follicle in the scalp and cultured on a 2D surface. However, over time, this feature is lost through prolonged culture and cell passaging. To this end, the 3D system offers a biomimetic platform to reproduce the hair follicle niche through micro‐fabrication. This system offers low attachment surfaces, allowing for self‐aggregation of the DP by exerting an external environmental stimulus. The self‐aggregation capacity of the DP is crucial for replicating the native state of the DP present within the hair follicles. This phenomenon allows us to reproduce the exact orientation of the cells within the hair follicle in vivo, by way of sequential seeding of different cell types to produce tri‐culture heterotypic spheroids to study their cell behaviour towards each other. The dimensions of the cell aggregates, and thus, the thickness of the shell layer, is dependent on the seeding concentration of these cells into the microwells. In this study, we used a concentration of 12 million cells/mL for the seeding of DP cells, yielding aggregate sizes of ~80‐100 µm in diameter. This phenomenon is due to the preferential adhesion between cells over the low attachment substrates/surfaces. Following aggregation, the cells undergo tissue compaction, a known quality and observation of cells grown on substrates promoting intercellular aggregation.^5^ Substrates that promote greater intercellular aggregation tend to produce aggregates which are rounder, more spherical and more compact in nature. The resulting sizes of the DP aggregates, therefore, correspond to the sizes of the DP embedded within the hair follicle.^18^ The seeding of HaCaT keratinocytes and HDF cells at subsequent intervals 24 hours apart at 24 million cells per ml yielded aggregates which were ~150 and 200 µm, respectively. The size of the human hair follicle is estimated to be around 200 µm in diameter.^18^ Therefore, these 3D constructs have demonstrated that it is able to recapitulate dimensions presented within in vivo hair follicles.

There was distinct compartmentalization of the DP cells and the HaCaT keratinocytes but there were no signs of migratory polarization when DP and HaCaT cells were co‐cultured together. However, in the 3D tri‐cultured spheroids, it was observed that the HDF cells exhibited anterior‐posterior polarity, a form of migratory polarization from the DP‐HaCaT co‐cultured spheroids. An interesting observation was made in the confocal images that the HDF cells were polarizing from the HaCaT keratinocytes and not from the DP cells, implying that the keratinocytes may play a role in ensuring compartmentalization between the DP cells and the surrounding HDF residing within the dermis. By ensuring compartmentalization, the keratinocytes have also assisted in maintaining the aggregative state of DP within the dermis, which is essential for hair follicle induction.

In conclusion, we have successfully developed a systematic in vitro approach by sequential seeding of dissociated DP cells, keratinocytes and HDF cells for producing large numbers of 3D heterotypic aggregates. This restored the spatial orientation among these cells in order to investigate the effects on their cellular compartmentalization. Using this tri‐culture model, we observed that keratinocytes may have a role in maintaining compartmentalization between the DP and surrounding HDF residing in the dermis, and therefore maintains the aggregative state of the DP cells, which is necessary for the hair follicle development and function. Future applications may harness similar models to prepare 3D tri‐cultures for studying cell‐cell behaviours in other physiological systems in vitro.

## CONFLICT OF INTERESTS

The authors state no conflict of interest.

## Data Availability

The data that support the findings of this study are available from the corresponding author upon reasonable request.
