# Supplementary material for: Keratinocytes maintain compartmentalization between dermal papilla and fibroblasts in 3D heterotypic tri‐cultures
Source: Cell Prolif. 2019 Aug 5;52(5):e12668. doi: 10.1111/cpr.12668 (PMC6797517; doi:10.1111/cpr.12668)
Supplement: Supplementary file 1 [file CPR-52-e12668-s001.docx]

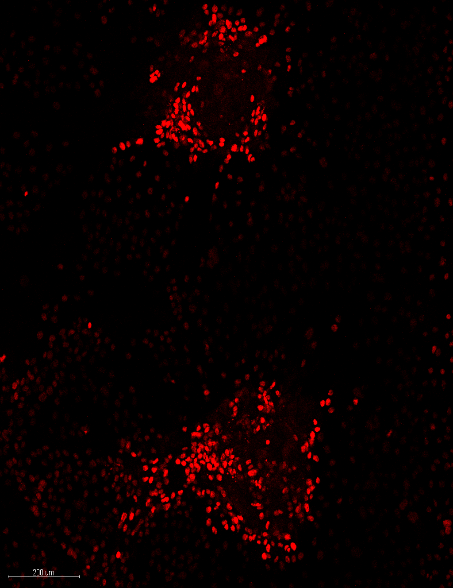

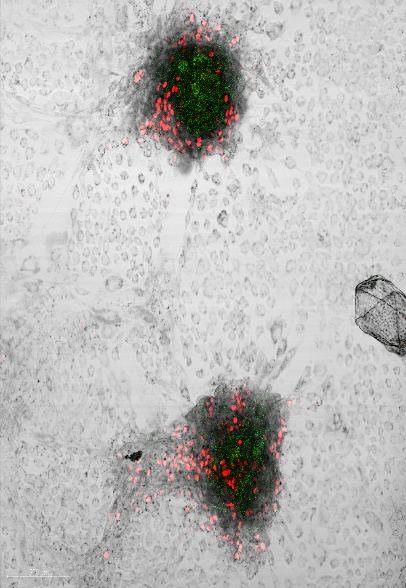

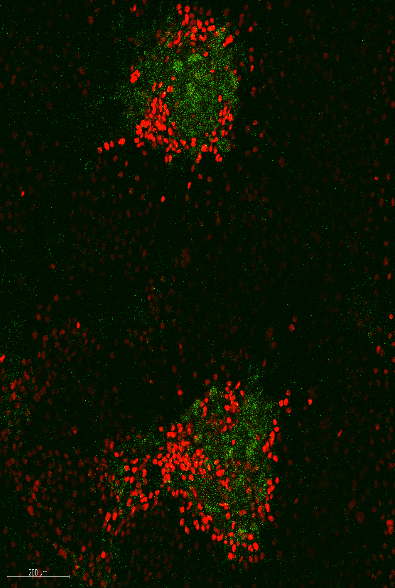

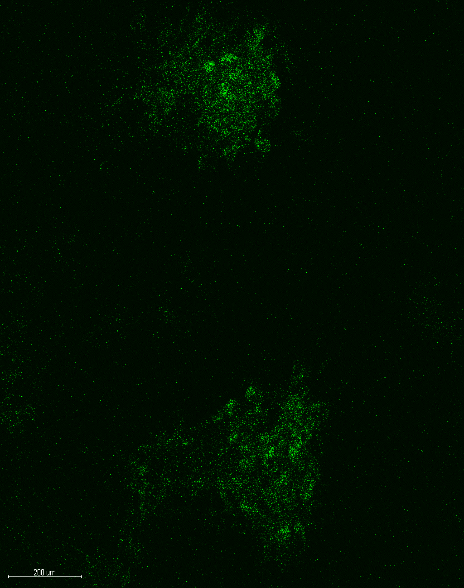


**Phase contrast**

**GFP DP & RFP HaCaT**

**GFP DP**

**RFP HaCaT**

**Supplementary figure 1**. **2D Distributions of GFP labelled DP cells and RFP labelled HaCaT keratinocytes in tri-cultures consisting of DP cells, HaCaT keratinocytes and HDF on Day 4 of incubation. (Scale bar = 200 µm)**
